# A Case Report of Endogenous Endophthalmitis Associated With a Pyogenic Liver Abscess Caused by Hypervirulent *Klebsiella pneumoniae*

**DOI:** 10.1002/iid3.70479

**Published:** 2026-08-24

**Authors:** Changjiang Liu, Jianting Song, Jiayuan Zhao, Boying Wei, Peng Wang, Chang Liu, Huatian Su, Xin Tong, Di Wu

**Affiliations:** ^1^ Department of Intensive Care Unit Jilin City Hospital of Chemical Industry Jilin Jilin China

**Keywords:** diabetes mellitus, endogenous endophthalmitis, hypervirulent *Klebsiella pneumoniae*, liver ultrasound, pyogenic liver abscess

## Abstract

**Objective:**

Pyogenic liver abscess (PLA) caused by hypervirulent *Klebsiella pneumoniae* (hvKp) may be complicated by endogenous endophthalmitis (EE) and pneumonia. We herein report a case of a Chinese male patient with PLA and bilateral EE caused by hvKp to highlight the severe clinical outcomes of this condition.

**Methods:**

This case report describes a 54 year‐old male with a history of poorly controlled diabetes mellitus (irregular oral medication and insulin therapy) who presented with a 7‐day fever prior to admission. He developed respiratory failure after hospitalization. *Klebsiella pneumoniae* was identified in sputum, blood, and liver puncture drainage fluid cultures, while vitreous fluid culture was negative. The patient was diagnosed with hvKp‐induced PLA complicated by pneumonia and bilateral EE.

**Results:**

Despite aggressive systemic and ocular treatment, the patient ultimately lost bilateral visual acuity and developed permanent blindness. Early liver ultrasound confirmed the presence of hvKp‐induced PLA.

**Conclusion:**

Patients with hvKp‐induced PLA are at high risk of developing EE, which carries a poor prognosis and may lead to irreversible blindness. Once suspected, prompt systemic and ocular intervention is critical to improve outcomes. This case underscores the value of early liver ultrasound in the diagnosis of hvKp‐induced PLA, which may facilitate timely management of life‐threatening complications.

## 1 Introduction

*Klebsiella pneumoniae* is currently classified into two types based on virulence characteristics: classical *Klebsiella pneumoniae* (cKP) and hypervirulent *Klebsiella pneumoniae* (hvKp) [1]. hvKp is an emerging pathogen with higher virulence than cKP. cKP is a Gram‐negative bacterium and a normal flora of the human nasal cavity and digestive tract. It usually causes pneumonia, urinary tract infections, and other infectious diseases in nosocomial and immunocompromised patients [1, 2]. In contrast, hvKp mainly infects healthy individuals in community settings and is characterized by metastatic infections, rapid disease progression, and poor prognosis. It is also an important pathogen causing community‐acquired pyogenic liver abscess (PLA) [1, 3]. hvKp was first identified in Taiwan, China [4]. In recent years, hvKp infections have also been reported in other Asian countries.

A serious and vision‐threatening complication of hvKp‐induced PLA is endogenous endophthalmitis (EE), which is associated with high mortality and severe visual loss. Recent studies have confirmed the strong association between hvKp infection and metastatic endophthalmitis, highlighting the importance of early recognition and intervention [5, 6, 7].

To date, clinical laboratories cannot easily differentiate hvKp from cKP. Therefore, it is difficult for clinicians to determine whether a patient is infected with hvKp or cKP, which may delay appropriate treatment for metastatic ocular complications. Previously, the hypermucoviscous phenotype was considered a useful test to distinguish hvKp from cKP, but this is no longer regarded as sufficiently reliable [8, 9]. Further basic and clinical studies are needed to improve the identification of hvKp. In some studies, hvKp strains are defined by the presence of key virulence biomarkers (e.g., *rmpA* and *iucA*–*iucD*), the presence of an hvKp‐specific virulence plasmid [9], the demonstration of high virulence in appropriate in vivo models, or highly suggestive clinical features (e.g., invasive infection in an otherwise healthy community host, especially with multiple sites of infection or metastatic spread).

Current reports indicate that hvKp can infect all ethnic groups, but Asians are the most commonly affected [10]. Other risk factors include male gender [10], diabetes mellitus [11], and exposure to ampicillin or amoxicillin within the previous 30 days [12].

The exact mechanism by which hvKp infects the liver remains unclear. Since PLA is the most common clinical manifestation of hvKp infection, it is hypothesized that the bacteria may reach the liver via the portal vein. Unlike cKP, hvKp can disseminate to multiple organs, including the eyes, brain, and spleen. Among all complications of hvKp‐induced PLA, EE is rare but one of the most severe. Once EE occurs, the prognosis is extremely poor, and patients may develop permanent blindness [13].

We herein report a case of a Chinese male patient with hvKp‐induced PLA, pneumonia, and bilateral EE. His condition was critical, complicated by respiratory failure and eventual complete visual loss. Through serial dynamic liver ultrasound observations, we further clarify the early ultrasound features of hvKp‐induced PLA.

## 2 Case Report

### 2.1 Case Presentation

A 54‐year‐old male patient presented to a local clinic with a high fever and was administered cephalosporin antibiotics. Nevertheless, his symptoms failed to improve, and he developed disturbance of consciousness on the seventh day of illness, leading to referral to our hospital.

On admission, his body temperature was 38.6°C and blood glucose was 36.1 mmol/L. He was initially admitted to the endocrinology department due to severe hyperglycemia. However, he rapidly progressed to respiratory failure and was transferred to the intensive care unit for endotracheal intubation and mechanical ventilation.

### 2.2 Preexisting Comorbidities and Medical History

The patient had an 8‐year history of diabetes mellitus (DM) and a 6‐year history of hypertension (HTN). He had no family history of liver disease, and he denied alcohol consumption or smoking.

### 2.3 Physical Examination

On examination, the patient had a body temperature of 38.6°C, respiratory rate (R) of 24 breaths/min, blood pressure (BP) of 112/75 mmHg, and heart rate (HR) of 112 beats/min. The patient's ocular appearance was normal; both pupils were equal in size (3 mm in diameter) with normal light reflexes, but visual acuity assessment was not feasible due to poor cooperation. No abnormalities were noted on cardiac and pulmonary auscultation; the abdomen was soft and flat. Mild pitting edema was present in both lower extremities.

### 2.4 Diagnostic Assessment

#### 2.4.1 Biochemical and Microbiology Examination

Laboratory findings on admission revealed a severe inflammatory status, as shown in Table 1. The bacterial “string test” was negative. Unfortunately, the detection of virulence genes was not conducted.

**TABLE 1:** Baseline laboratory data of the patient.

Hematologic test	
White blood cells	26.7 × 10^9^/L
Neutrophil	94.5%
Lymphocyte	2.5%
Monocyte	1.10%
Eosinophil	1.50%
Basophil	0.40%
Red blood cells	6.34 × 10^12^/L
Hemoglobin	195 g/L
Platelet count	15 × 10^9^/L
Coagulation	
PT	17.9 s
APTT	36.5 s
Chemistry	
AST	1349.69 U/L
ALT	812.54 U/L
LD	1597.75 U/L
ALP	598 U/L
γ‐GTP	226.54 U/L
Total bilirubin	198.82 µmol/L
Total protein	61.45 g/L
Albumin	29.28 g/L
BUN	25.90 mmol/L
Creatinine	228.80 µmol/L
Sodium	138 mmol/L
Potassium	4.4 mmol/L
Chloride	100 mmol/L
CRP	142 mg/L
PCT	> 100 ng/mL
Glucose	36.1 mmol/L
HBs‐Ag	(−)
Laboratory culture	
Blood	*Klebsiella pneumoniae*
Vitreous	(−)
Drainage fluid from the liver puncture	*Klebsiella pneumoniae*
Sputum	*Klebsiella pneumoniae*

Abbreviations: ALP, alkaline phosphatase; ALT, alanine aminotransferase; APTT, activated partial thromboplastin time; AST, aspartate aminotransferase; BUN, blood urea nitrogen; CRP, C‐reactive protein; γ‐GTP, γ‐glutamyl transpeptidase; PCT, procalcitonin; PT, prothrombin time activity.

#### 2.4.2 Imaging Examination

**FIGURE 1:**
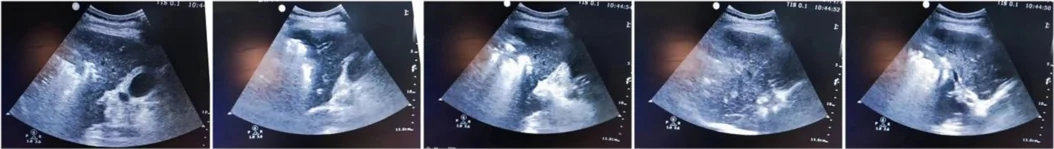
The liver ultrasound of the patient on the first and second day of admission showed strong flaky echoes can be seen in the liver, and the posterior structure is unclear.

**FIGURE 2:**
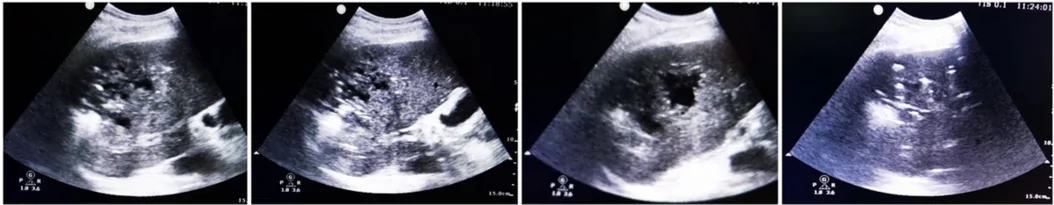
The liver ultrasound of the patient on the third day of admission showed that the right lobe of the liver was heterogeneous with a slightly stronger echo (partial liquefaction), about 91 × 92 mm in size.

**FIGURE 3:**
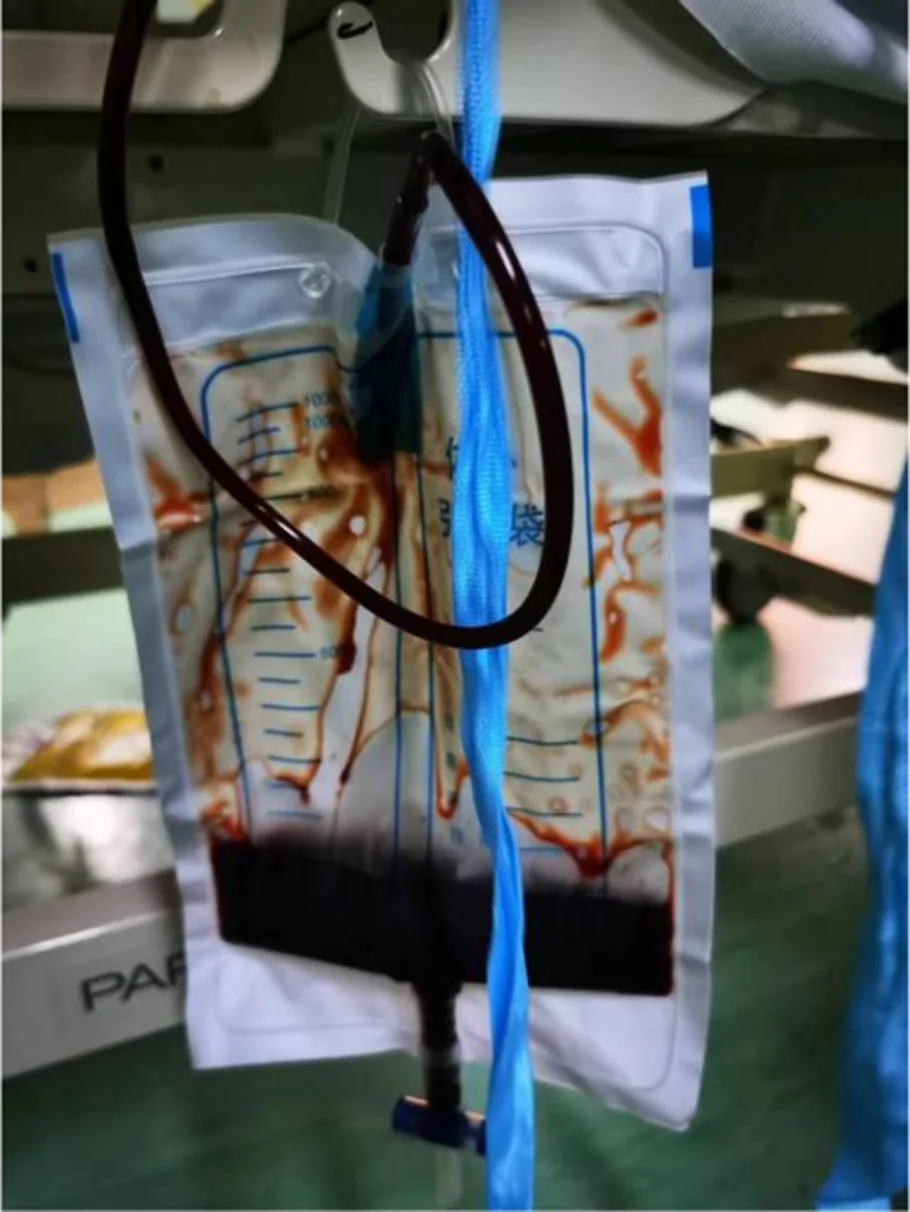
Liver puncture drainage resulted in dirty blood liquid, and culture of the drainage fluid grew *Klebsiella pneumoniae*.

**FIGURE 4:**
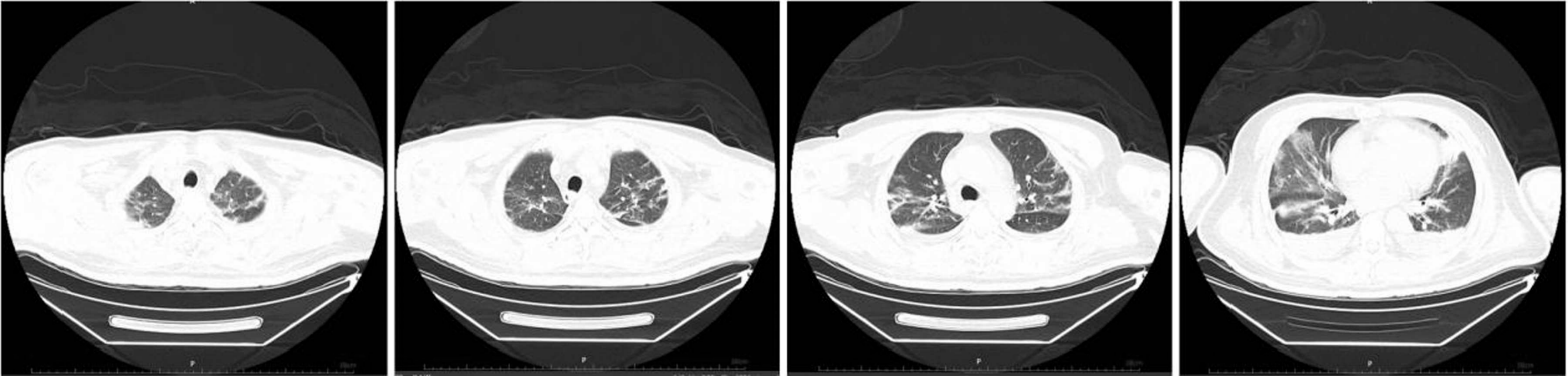
The chest CT of the patient on the eighth day of admission showed a double lung infection.

**FIGURE 5:**
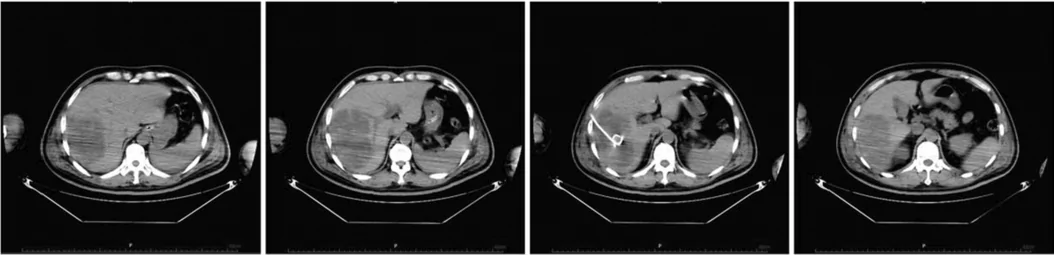
The abdominal CT of the patient on the eighth day of admission showed a slightly low‐density image of the right lobe of liver, about 113 × 73 mm in size. And the liver puncture catheter placement for drainage.

On the first and second days of admission, abdominal ultrasound of the patient showed flaky strong echoes in the liver with unclear posterior structures (Figure 1). On the third day, abdominal ultrasound revealed heterogeneous echo in the right hepatic lobe with slightly increased echogenicity (partial liquefaction), measuring approximately 91 × 92 mm with ill‐defined boundaries (Figure 2). Given that the abdominal ultrasound indicated liver abscess liquefaction, we performed percutaneous puncture and drainage for the liver abscess, and the drained fluid was dirty, non‐coagulable blood (Figure 3). On the eighth day of admission, computed tomography (CT) scans of the head, chest, and entire abdomen were performed. Head CT showed no abnormalities, while chest CT indicated bilateral pulmonary infection (Figure 4). Abdominal CT demonstrated a slightly hypodense lesion in the right hepatic lobe, approximately 113 × 73 mm in size (Figure 5). On the ninth day, ocular ultrasound of the patient revealed bilateral intravitreal hyperechoic bands, and retinal detachment was suspected.

### 2.5 Treatment and Outcomes

After admission, the patient received antibiotic therapy, blood glucose control, mechanical ventilation, and symptomatic treatment. Empiric antimicrobial therapy was initiated with biapenem 0.3 g intravenously every 12 h and vancomycin 0.5 g intravenously every 12 h. On the third day, liver abscess puncture drainage was performed. On the fourth day, the patient's admission sputum and two blood culture results were positive for *Klebsiella pneumoniae*; the drug sensitivity test showed sensitivity to biapenem, so vancomycin was discontinued, and biapenem was continued as in‐hospital anti‐infective therapy. On the fifth day, the culture of liver puncture drainage fluid also yielded *Klebsiella pneumoniae*. Since the patient had been on invasive mechanical ventilation since admission, he was unable to express discomfort complaints. On the eighth day, the patient was weaned off the ventilator. On the ninth day, the patient complained of bilateral eye pain, and an ophthalmology consultation was requested. On physical examination, the ophthalmologist found that the patient had uncertain light perception in both eyes, high intraocular pressure, mild mixed congestion in both eyes, smooth corneal reflex, suspected yellowish‐white aqueous humor, round and centered pupils with present direct and indirect light reflexes, and grayish‐yellow reflection behind the lens in the pupillary area. Funduscopy showed vitreous opacity with invisible retina. Diagnosis: binocular endophthalmitis, vitreous opacity, and binocular glaucoma. Advice: Continue systemic anti‐infection and perform binocular vitreous aspiration + intravitreal drug injection. The ophthalmologist treated the patient accordingly, but the patient's ocular condition did not improve after treatment. On the 11th day, the ophthalmologist performed a second intravitreal drug injection. On the 13th day, the patient was transferred to a higher‐level hospital for further treatment on January 27, 2024. Three months after transfer, we were informed by telephone that the patient had eventually lost his vision.

## 3 Discussion

In Europe and the United States, the primary pathogen of PLA is *Escherichia coli* [3]. However, in recent years, an increasing number of cases of PLA caused by *Klebsiella pneumoniae* have been reported, most of which occur in Asian populations. This may be associated with the higher prevalence of intestinal colonization with K1/K2 serotype *Klebsiella pneumoniae* in Asian individuals [14]. At present, the initial route by which hvKp invades the liver remains unclear [1]. Patients infected with hvKp typically lack obvious skin or mucosal lesions, and liver abscess is the most common clinical manifestation [1]. Therefore, it is inferred that the bacteria may enter the portal vein from the intestine and subsequently cause PLA. PLA caused by hvKp can be complicated by pneumonia, endophthalmitis, and meningitis, with a poor overall prognosis. Delayed diagnosis of endophthalmitis is associated with a high risk of permanent blindness.

Endophthalmitis is classified into exogenous endophthalmitis and EE based on the route of infection [15]. EE arises from hematogenous spread of microorganisms from extrapulmonary sites to the eye. The case reported herein involves EE secondary to hvKp infection. EE is not exclusive to *Klebsiella pneumoniae* infection and can also be caused by other pathogens, including *E. coli* and fungi. Due to the blood–retinal barrier, systemic anti‐infective therapy alone is often insufficient to treat ocular involvement. To date, few randomized controlled trials have evaluated strategies for preventing endophthalmitis [16].

Given that EE is a localized manifestation of systemic infection and relatively rare, it is frequently overlooked by clinicians, leading to delayed treatment. Therefore, in patients with poorly controlled diabetes mellitus, severe systemic infection, or a deteriorating clinical state, clinicians should consider the possibility of metastatic infection involving other organs [11]. Combined with current literature reports, the eye is an important organ that should not be neglected in such patients [17]. Patients with EE typically present acutely: 90% report decreased visual acuity, 50% experience ocular pain, 35% develop hypopyon, and 33% have vitritis [18]. The patient in our report presented with decreased visual acuity and ocular pain. On ultrasonography, PLA typically manifests as hypo‐ or hyperechoic lesions with internal debris, while CT shows non‐enhancing hypodense lesions with rim enhancement [19, 20]. Inexperienced ultrasonographers may misdiagnose these findings as intrahepatic bile duct lesions, highlighting the need for increased awareness and further study of the ultrasonic features of PLA.

For the management of PLA, no formal guidelines or randomized controlled trials have established optimal empiric antimicrobial regimens or treatment durations. Empiric therapy should be guided by the isolated pathogen, antimicrobial susceptibility patterns, and local epidemiological data, with adjustments based on culture results [3]. The optimal duration of antimicrobial therapy is typically determined by the severity of infection and the patient's clinical response to initial treatment [20].

In the treatment of EE, intravitreal antibiotic injection or vitrectomy may be performed. Empiric intravitreal injection of ceftazidime combined with vancomycin is a common first‐line approach [18]. If fungal infection is suspected, intravitreal injection of amphotericin B or voriconazole may be considered [13]. The prognosis of EE is primarily associated with the causative pathogen [15]. Gram‐negative bacterial endophthalmitis carries a particularly poor prognosis. Visual loss is common in endophthalmitis, but final visual acuity may not be determined until weeks or months after complete resolution of inflammation. Thus, aggressive intervention to improve ocular outcomes is warranted, regardless of the severity of ocular involvement in the early stages of the disease.

### 3.1 Limitations

This study has several limitations. First, it is a single‐case report, which limits the generalizability of our findings to broader patient populations. Second, we did not perform molecular characterization of the hvKp strain (e.g., detection of virulence genes such as *rmpA* or *iucA*), which would have provided additional insights into the pathogenicity of the isolate. Third, long‐term follow‐up data on the patient's systemic and ocular outcomes were not available. Future large‐scale, multicenter studies are needed to validate the role of early liver ultrasound in identifying hvKp‐induced PLA and preventing metastatic complications such as EE.

## 4 Conclusion

hvKp‐induced PLA can lead to metastatic infection of multiple organs, and EE is associated with a high risk of permanent blindness. This case highlights the value of early liver ultrasound in identifying hvKp‐induced PLA, which may facilitate timely intervention to prevent severe metastatic complications. Future research should focus on developing early screening strategies for high‐risk patients (e.g., those with poorly controlled diabetes mellitus) to reduce the incidence of devastating complications such as bilateral EE.

## Author Contributions

**Changjiang Liu:** conceptualization, data curation, investigation, supervision, writing – original draft, writing – review and editing. **Jianting Song:** conceptualization, data curation, investigation, writing – original draft, writing – review and editing. **Jiayuan Zhao:** investigation, data curation, writing – review and editing. **Boying Wei:** investigation, data curation, writing – review and editing. **Peng Wang:** investigation, data curation, writing – review and editing. **Chang Liu:** investigation, writing – review and editing, data curation. **Huatian Su:** investigation, writing – review and editing, data curation. **Xin Tong:** investigation, writing – review and editing, data curation. **Di Wu:** supervision, conceptualization, project administration, writing – review and editing, resources.

## Funding

The authors have nothing to report.

## Ethics Statement

Written informed consent was obtained from the patient for publication of this case report.

## Consent

Written informed consent was obtained from the patient for publication of this case report.

## Conflicts of Interest

The authors declare no conflicts of interest.

## Data Availability

The data that support the findings of this study are available from the corresponding author upon reasonable request.
